# The Evaluation of Clinical Signs and Symptoms of Malignant Tumors Involving the Maxillary Sinus: Recommendation of an Examination Sieve and Risk Alarm Score

**DOI:** 10.3390/healthcare11020194

**Published:** 2023-01-09

**Authors:** Atif Bashir, Zafar Ali Khan, Afsheen Maqsood, Namdeo Prabhu, Muhammad Mudassar Saleem, Bader K. Alzarea, Rakhi Issrani, Shammas Raza Khan, Naseer Ahmed, Maria Shakoor Abbasi, Anand Marya, Mohammed Ghazi Sghaireen, Artak Heboyan

**Affiliations:** 1 Oral and Maxillofacial Surgery Unit, Cork University Hospital, T12 DC4A Cork, Ireland; 2 Oral and Maxillofacial Surgery Department, Dental Section, King Saud Hospital, Unaizah 56437, Saudi Arabia; 3 Department of Oral & Maxillofacial Surgery and Diagnostic Sciences, College of Dentistry, Jouf University, Sakaka 72345, Saudi Arabia; 4 Department of Oral Pathology, Bahria University Dental College, Karachi 74400, Pakistan; 5 Consultant Oral and Maxillofacial Surgeon Yanbu General Hospital, Yanbu 46411, Saudi Arabia; 6 Department of Prosthetic Dental Science, College of Dentistry, Jouf University, Sakaka 72345, Saudi Arabia; 7 Department of Preventive Dentistry, College of Dentistry, Jouf University, Sakaka 72345, Saudi Arabia; 8 Oral and Maxillofacial Unit, King Salman Hospital, Hail 55471, Saudi Arabia; 9 Prosthodontic Unit, School of Dental Sciences, Health Campus, Universiti Sains Kota Bharu, Kubang Kerian 16150, Malaysia; 10 Department of Prosthodontics, Altamash Institute of Dental Medicine, Karachi 75500, Pakistan; 11 Department of Orthodontics, Faculty of Dentistry, University of Puthisastra, Phnom Penh 12211, Cambodia; 12 Center for Transdisciplinary Research, Saveetha Dental College, Saveetha Institute of Medical and Technical Science, Saveetha University, Chennai 600077, India; 13 Department of Prosthodontics, Faculty of Stomatology, Yerevan State Medical University after Mkhitar Heratsi, Str. Koryun 2, Yerevan 0025, Armenia

**Keywords:** epistaxis, exophthalmos, maxillary sinus, malignant, squamous cell carcinoma

## Abstract

This observational study was conducted to evaluate the clinical signs and symptoms of maxillary sinus tumors and to propose a clinical examination sieve and a unique risk alarm score to be used for timely patient referral and vigilance. The study consisted of 70 patients between 20 to 82 years of age from both sexes. The clinical information gained was collected from the upper dentoalveolar segment, orbit, and nasal sites. Regarding the early clinical sign and symptoms of patients, nasal obstruction was found in 67 patients (95.7%), facial swelling in 69 patients (98.6%), paresthesia in 41 patients (58.6%), and epistaxis in 50 patients (71.4%). Furthermore, in terms of the late signs and symptoms, a palpable mass in buccal sulcus was observed in 65 (92.9%) of the patients, lymphadenopathy in 24 (34.3%), paresthesia in 38 (54.3%), and diplopia in 22 (31.4%). Furthermore, general sign and symptoms like exophthalmos was present in 35 patients (50%), anosmia was observed in 37 patients (52.9%), and oroantral fistula was noted in 37 patients (55.9%). Additionally, 67 (95.7%) of the patients complained of nasal obstruction. Similarly, facial asymmetry was observed in 69 (98.6%) of the patients and double vision was observed in 24 (34.4%). Tumors of the maxillary sinus have a very insidious course of spread and uncertain clinical signs and symptoms. What makes diagnosis worse is the fact that the symptoms of these tumors are so well hidden in the sponge-like nature of the midfacial region that they are easily misinterpreted by patients. Therefore, diagnoses must be made early, dentists must be vigilant, and patients must be fully investigated at the slightest suspicion of a tumor, albeit benign.

## 1. Introduction

A malignant tumor of the maxillary sinus (MTMS), which comprises squamous cell carcinoma, adenoid cystic carcinoma, adenocarcinoma, and rhabdomyosarcoma, is a rare clinical entity that poses significant diagnostic and management challenges for physicians. Patients with these tumors often present with locally advanced disease near vital structures [1]. The average volume of the fully developed human maxillary sinus is approximately 25 mL in males and 15 mL in females. This volume may potentially allow an MTMS to grow to a significant size before the clinical signs and symptoms develop [2].

The presence of air-filled spaces permits the silent growth of an MTMS, which produces few signs and symptoms until a considerable volume has been reached [3]. This air-filled nature and the deep position of the structures involved is the reason that an MTMS is difficult to diagnose during its early stages, even when an adequate diagnostic imaging tool is used [4,5].

The clinical signs and symptoms of an MTMS fall into five categories: oral, nasal, ocular, facial, and auditory. The oral symptoms include a widened alveolus, dental pain, an obvious palatal mass, and ulceration or a non-healing wound (such as an extraction socket). The nasal symptoms include unilateral nasal obstruction, epistaxis, anosmia, nasal drainage, and hyponasal speech. Orbital or ocular symptoms include proptosis, eyelid edema, diplopia secondary to extraocular muscle involvement, epiphora resulting from obstruction or destruction of the lacrimal drainage apparatus, or visual loss from direct involvement of the optic or oculomotor nerves at the orbital apex. Facial symptoms include loss of definition of the nasolabial fold of the involved side, facial asymmetry, an obvious cheek mass, cutaneous fistula, facial edema, and pain. In addition, hypoesthesia of the cheek may also occur secondary to invasion of the infraorbital nerve. Hearing loss can develop from nasopharyngeal extension of the tumor, which can lead to eustachian tube obstruction or dysfunction and serous effusion. The symptoms that result from the posterior extension of an MTMS include severe, deep-seated pain due to invasion of the skull base, trismus due to pterygoid muscle invasion, and cranial neuropathies [2,4,5].

Lee et al. [6] proposed that the average delay between the onset of symptoms related to an MTMS and receipt of a definitive diagnosis is approximately eight months. Patients usually overlook symptoms including nasal blockage, tooth loosening, and pain because they assume that they could be due to other disease conditions such as sinusitis, periodontal diseases, and facial pain, respectively. Hence, treatment of these diseases continues for months and years.

The timely diagnosis of an MTMS, which is solely dependent on knowledge and identification of early and late clinical signs and symptoms, is essential for the appropriate management of the condition and improves patient prognosis [7]. Delayed management is not only related to the neglecting of symptoms by the patients, but also due to misdiagnosis by general practitioners, otorhinolaryngologists, dentists, and occupational health physicians, the latter of which is partly due to the impossibility of direct inspection and palpation of the maxillary sinus in comparison to the oral cavity [8]. The limited scientific data, diagnostic rationale, and lack of treatment guidelines in these fields can be explained by the rarity of the MTMS [8]. An earlier diagnosis, and consequently a shorter time interval from first symptom to diagnosis, is assumed to correlate with a better outcome. The scientific data available on the prognostic significance of the “time interval from first symptom to diagnosis” in relation to maxillary sinus tumors is limited [9]. Therefore, there is a need to determine early and late clinical signs and symptoms of an MTMS, so that dentists, general medical practitioners, otolaryngologists, and maxillofacial surgeons can become vigilant and aware of maxillary sinus tumors and can arrive at the correct diagnosis as early as possible. Thus, the aim of this study was to evaluate the clinical signs and symptoms of maxillary sinus malignant tumors and to propose a clinical examination sieve along a unique risk alarm score that can be used for timely referral and increased vigilance.

## 2. Materials and Methods

### 2.1. Study Setting and Sample Size

This descriptive observational study was conducted at a tertiary care hospital, specifically the oral and maxillofacial surgery department of King Edward medical university and Mayo hospital, Lahore, Pakistan, over a period of three years from January 2016 to December 2019. The sample size of 70 patients was calculated with a 95% confidence level and 5% margin. The power of the test was 80. The calculation was done by considering the expected percentage of anosmia (i.e., 12%) in patients with maxillary sinus malignant tumors.

### 2.2. Ethical Consideration and Participants Recruitment

This study was approved by the institutional review board (2047/RC/KEMU). The study was designed according to the STROBE guidelines (Appendix A). Patients with malignant tumors of the maxillary sinus who fulfilled the inclusion and exclusion criteria were enrolled in the study after providing written informed consent. A non-probability purposive sampling technique was employed to recruit participants. All patients of both genders over the age of 18 years and with biopsy-proven malignant tumors of the maxillary sinuses were included. Those patients who previously had surgery for their MTMS or a combination of surgery with radiotherapy or chemotherapy were excluded from the study.

### 2.3. Data Collection

The data were collected by the principal investigator (A.B.) and co-investigator (Z.A.K). Patients who attended the oral and maxillofacial surgery outpatient department first underwent a history and clinical examination. Demographic information including each patient’s age, sex, and address was recorded. The patients were assessed for the following signs: exophthalmos, anosmia, oroantral fistula palatal tumor, lymphadenopathy, nasal obstruction, nasal bleeding, facial asymmetry, cheek swelling, and double vision (diplopia). Additionally, the patients were categorized into groups based on whether they were experiencing late or early clinical signs and symptoms. Clinical signs and symptoms were considered ‘early’ if the patient sought medical consultation within the first one or two months after their initiation and were considered ‘late’ if the patient sought medical consultation for their symptoms three to eight months after the initial visit. All information was entered on the proforma (Supplementary File (Annexure S1)).

### 2.4. Statistical Analysis

The data were analyzed in SPSS-25. The age was presented as mean and standard deviation. Gender, early and late clinical signs and symptoms (exophthalmos, anosmia, oroantral fistula, mass in the buccal sulcus, lymphadenopathy, nasal obstruction, nasal bleeding, asymmetry, cheek swelling, and double vision) were presented as frequencies and percentages. The Chi-square test was used to analyze the association between clinical signs and symptoms of maxillary sinus malignancy and gender. The test was also used to detect any association between occupation and history of exposure to hazards as well as with the type of maxillary sinus tumor. Results with a *p*-value ≤ 0.05 were considered to be statistically significant.

## 3. Results

The mean age of the patients in this study was 53.82 ± 15.81 years. The minimum age of the patients was 20 years and the maximum age was 82 years. Regarding the gender distribution, 47 (67.1%) of patients who participated in this study were male, while 23 (32.9%) were female. The male-to-female ratio was almost two-fold, i.e., 2.04:1. Among the gross clinical signs and symptoms, exophthalmos was seen in 35 patients (50%), anosmia (inability to smell) was seen in 37 patients (52.9%), oroantral fistula was seen in 39 patients (55.7%), a palpable mass in the buccal sulcus was seen in 66 patients (94.3%), and lymphadenopathy was seen in 25 patients (35.7%). Furthermore, nasal obstruction was present in 67 patients (95.7%) and nasal bleeding/discharge was present in in 56 patients (80%). Facial asymmetry and cheek swelling was observed in 69 patients (98.6%). Lastly, double vision was found in 24 patients (34.3%). There was no significant difference between the sexes in terms of the clinical signs and symptoms of maxillary sinus malignancy (Chi-square test; *p* > 0.05). This indicates that the clinical sign and symptoms of this condition were equally distributed among males and females (Table 1).

**Figure 1:**
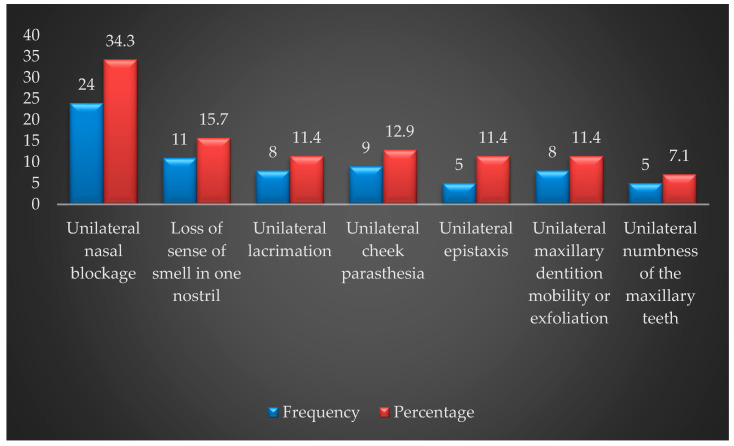
Characteristics of the early clinical signs and symptoms of maxillary sinus tumors (*n* = 70).

Furthermore, of the patients who presented with early clinical signs and symptoms, nasal obstruction was observed in 24 (34.3%), anosmia was observed in 11 (15.7%), paresthesia was observed in nine (12.9%), numbness of the teeth was observed in five (7.1%), and epistaxis was observed in five (11.4%) (Figure 1). Of the patients with late clinical signs and symptoms, a palpable mass in the buccal sulcus was observed in 65 (92.9%), lymphadenopathy was observed in 24 (34.3%), paresthesia was observed in 38 (54.3%), and diplopia was observed in 22 (31.4%).

A significant association was found between having a history of hazards exposure and both SCC (Chi-square test; *p* = 0.001) and ACC (Chi-square test; *p* = 0.021). This suggests that the prevalence of these maxillary sinus tumors varies between patients belonging to different occupations. Among the hazards that were considered to be a possible cause of maxillary sinus malignancy, firsthand or secondhand tobacco smoke exposure (27 patients (38.57%)) was the most prevalent, followed by wood dust (26 patients (37.14%)). Moreover, as shown in Table 2, no significant associations were found between exposure to the various hazards analyzed in this study and maxillary sinus tumors (Chi-square test; *p* > 0.05).

Regarding patients’ occupations, maxillary sinus carcinoma was most prevalent in carpenters (18 patients (25.71%)), urban housewives (11 patients (15.71%)), wood cutters (8 patients (11.42%)), and rural housewives (8 patients (11.42%)).

**Figure 2:**
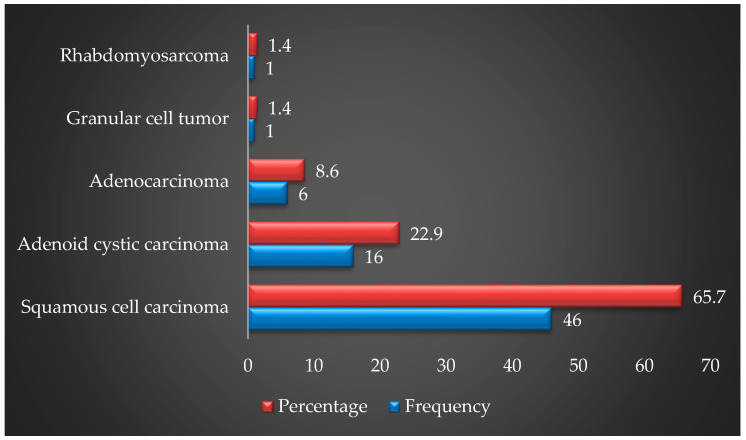
Distribution of the types of maxillary sinus tumors in patients (*n* = 70).

As presented in Figure 2, squamous cell carcinoma, which was the most common malignancy, was encountered in 46 patients (65.7%), while adenoid cystic carcinoma was found in 16 patients (22.9%).

## 4. Discussion

Malignant tumors of the maxillary sinus are difficult to diagnose during their early stages of development due to the complex anatomic structure of the maxillary sinus. The identification of both early and late signs and symptoms of this condition can help in the timely diagnosis and appropriate management of an MTMS, which can subsequently improve the patient’s prognosis [7,10]. Early signs and symptoms are usually present when the tumor has invaded only the paranasal sinuses. Initially, these symptoms are non-specific, i.e., unilateral nasal blockage, mucopurulent rhinorrhea, and epistaxis [9]. Patients often neglect these symptoms for a long time, especially those who have had chronic exposure to wood dust and/or have experienced symptoms for many years.

Tobacco smoking is an established risk factor for maxillary sinus malignant tumors [8]. In 2018, a group from Spain (Natalia et al. [8]) reported a case series of maxillary sinus carcinoma in 24 patients and noted that all their patients were smokers. Tobacco smoking is considered to be directly associated with an elevated risk of paranasal sinus malignancies, and a prolonged duration of smoking and higher number cigarettes smoked per day doubles this risk [9,10]. Twenty-seven (38%) patients in our study that presented with maxillary sinus malignancies had a history of tobacco smoking. Apart from vast socio-economic differences between the populations of Spain and Pakistan, his variation may be explained by a difference in the prevalence of daily tobacco cigarette smoking between these countries. According to the 2019 WHO report on the global tobacco epidemic, in 2017, the daily smoking prevalence among adults in Spain was 24%, while in Pakistan an estimated 13% of the adult population smoked tobacco cigarettes daily [11].

Wood dust has been classified as carcinogenic to humans by the International Agency for Research on Cancer (IARC) [7]. The risk of maxillary sinus and nasal cancer is reported to be strongly associated with previous exposure to wood dust [12]. This may explain why a higher percentage of clinical symptoms in our study presented as early features. In our study, 26 patients (37%) had a history of exposure to wood dust, and among them, 18 (69%) were carpenters by profession, while eight (30%) were timber wood cutters. In a study conducted between 2007 and 2009 that involved 934 workers exposed to wood dust in Brittany, Jegoux F et al. [7] reported that 25% of the participants had a blocked nose, 18% had rhinorrhea and sneezing symptoms, and 9.9% had epistaxis. In our study, nasal obstruction was found in 67 patients (95.7%) and facial swelling was found in 69 patients (98.6%). Furthermore, paresthesia was observed in 41 patients (58.6%), epistaxis was observed in 50 patients (71.4%), and anosmia was observed in 37 patients (52.9%). The difference in the prevalence of the different clinical signs and symptoms could be due to the small sample size and exposure to other risk factors like coal dust, spray paint/chromium, and cow dung smoke reported in the current study.

Biomass smoke has been termed a ‘probable carcinogen’ (Group 2a) by The International Agency for Research on Cancer (IARC), while coal (used as domestic fuel) has been termed carcinogenic to humans (Group 1) [13]. Similarly, domestic coal smoke is a significant risk factor for the development of lung cancer [14]. Daily and prolonged exposure to aerial dust particles that are released during the combustion of biomass fuels has been associated with an elevated risk of acute infections of the respiratory tract, chronic obstructive pulmonary disease, and malignancies of the lungs. In Pakistan, approximately 70% of the population lives in rural areas, with 94% of rural homes and 58% of urban homes depending upon biomass fuels, including cow dung [15]. Eleven percent of our patients who presented with maxillary sinus malignancies had a history of prolonged exposure to smoke from wood or cow dung used as cooking fuel in rural Pakistan. Although these types of smoke have being labeled as probable carcinogens by the IARC based on their association with the development of lung cancer, to the best of our knowledge, there is no data available regarding the association of biomass smoke from wood and cow dung with maxillary sinus carcinomas.

Spray paint and its constituents (silica, chromium, lead, isocyanates, and iron oxide) are associated with countless health hazards like asthma and bronchitis. In particular, prolonged chromium exposure has been especially associated with a higher risk of bronchogenic and sinonasal cancers [16,17,18]. Three (4.2%) of our patients presenting with maxillary sinus carcinoma were professional “automobile spray painters”. In 2013, Choi et al. [19] reported the first case of a spray painter who developed malignant fibrous histiocytoma of the maxillary sinus following long-term exposure to chromium, nickel, and formaldehyde, implying that these agents are probable causal agents.

When the tumor extends beyond the boundaries of the sinus, in addition to sinus-related symptoms, certain neurological symptoms, including headache; anesthesia in the territory of the trigeminal nerve; ophthalmological symptoms, such as exophthalmos, recurrent conjunctivitis, and diplopia; or dental symptoms including pain and mobility of the maxillary teeth, may also become evident [20,21,22,23]. In a study conducted by Andrade et al. [24] from 1997–2006, the most common clinical signs and symptoms reported among patients with adenoid cystic carcinoma of the maxillary sinus were facial swelling (33.3% of patients), mouth swelling (29.2% of patients), and nasal obstruction (12.5% of patients). The most frequently recorded late signs and symptoms were tumor mass (87.5% of patients), pain (50% of patients), nasal obstruction (25% of patients), and epistaxis (20.8% of patients). The authors also observed that all patients had more than one sign and/or symptom, with the combination of facial swelling and pain being present in most (41.7%) of the cases, all of which were cases of advanced disease. In the current study, we observed facial swelling in 69 patients (98.6%), while an intraoral palpable mass in the buccal sulcus was seen in 65 patients (92.9%), and nasal obstruction was seen in 67 patients (95.7%). Of the patients with advanced disease, lymphadenopathy was seen in 24 (34.3%), paresthesia was seen in 38 (54.3%), and diplopia was seen in 22 (31.4%) as a late clinical sign or symptom. This finding is dissimilar to that of Andrade et al. [24], which is possibly due to variations in the sociodemographic factors and long-term follow-up of the participants, especially with respect to disease progression and the number of risk factors present.

Evidence suggests that the MTMS has a variable biological behavior and mode of presentation. For example, some authors have reported a 40.7% incidence of cervical nodal metastasis at first presentation [25,26]. In another study, Qureshi et al. [27] reported that the most common symptoms were facial swelling (73.8%), oral symptoms (26.2%), epistaxis (21.4%), and nasal obstruction (23.8%); increased lacrimation and proptosis were present in three and two of their patients, respectively. Furthermore, the most common clinical findings in their patients were facial mass in 33 patients (78.6%), intraoral mass in nine patients (21.4%), intranasal mass in 10 patients (23.8%), palpable neck mass in two patients (4.7%), and trismus in one patient. None of their patients had distant metastases at the time of diagnosis, however, six patients developed distant metastases at follow-up. Interestingly, the percentages in the current study are higher for almost all the signs and symptoms. This could be attributed to patient characteristics (i.e., drug abuse, unemployment, and poor socioeconomic status), tumor characteristics (i.e., rapid growth and poor prognostic histology), or other causes such as the poor medical facilities in rural areas.

For most primary care doctors, including general practitioners, dentists, and occupational health physicians, an MTMS may be a once-in-a-career diagnosis and can easily be missed [28]. A trained physician can arrive at an early diagnosis only if they possess specific knowledge and are able to identify the early signs and symptoms of an MTMS, both of which are essential for appropriately managing the condition and improving the prognosis of patients [7]. Delayed management is often related to patients neglecting such early symptoms because they do not consider them to be serious in nature and therefore, do not seek any early consultations for their symptoms [29]. Due to the unfeasibility of direct inspection and palpation of the maxillary sinus in comparison to the oral cavity, an MTMS may remain underdiagnosed and misdiagnosed by general practitioners, otorhinolaryngologists, dentists, and occupational health physicians [8]. The early signs and symptoms of an MTMS are key indicators that should act as triggers for early referral and timely diagnosis [28]. In general, unilateral nasal symptoms (for example, unilateral nasal obstruction), especially if the symptoms are grouped (for example, unilateral nasal obstruction, unilateral bloodstained discharge, and unilateral pain or orbital symptoms), should be seen as red flags and must raise the suspicion of an MTMS [7,30,31].

The identification of the early symptoms of an MTMS can help clinicians distinguish between benign or malignant conditions. However, it is vital to note if the symptoms are unilateral. Issues such as unilateral nasal blockage will require ear, nose, and throat (ENT) examination irrespective of the cause because benign conditions such as rhinosinusitis do not usually present unilaterally. Rather than treating the symptom with topical steroid sprays or decongestants in primary care, it would be more appropriate to refer a patient with unilateral sinonasal symptoms at the earliest [28,29]. The ulceration of the palatal or buccal oral mucosa in the maxilla; enlargement of the maxillary teeth-bearing alveolar bone or palate; creation of an opening between the oral cavity and maxillary sinus after extraction of a hypermobile maxillary tooth; extemporaneous exfoliation of sound permanent dentition; or unilateral, unexplained mobility of permanent teeth that cannot be attributed to any other cause; including periodontal disease [32] (which is often generalized and bilateral), unexplained and permanent numbness of maxillary dentition, or palatal or buccal mucosa; should act as sufficient trigger to cause a general dentist to suspect a maxillary sinus pathology and prompt a subsequent referral to the maxillofacial surgeon [8,29,33].

The findings of this study possess enormous clinical relevance and significance for primary care doctors, including general practitioners, dentists, and occupational health physicians, who can play a vital role in the timely diagnosis, appropriate management, and subsequently, an improved patient prognosis, as discussed previously.

Based on the findings of this study, we propose a clinical examination sieve and a risk alarm score to help guide general medical and dental practitioners in identifying the symptoms of an MTMS that will help in the early diagnosis and timely referral of patients to an expert maxillofacial and ENT surgeon in tertiary care centers, as described in Supplementary Table S1 and Figure S1.

## 5. Limitation

The limitation of our study was the small sample size. The data were collected at a single point in time and did not consider follow-up, treatment outcome, and initial disease progression. Moreover, the clinical examination sieve and risk alarm score were proposed based on the findings of this study, however, we were unable to validate both of these diagnostic tools during the course of our research. Therefore, we recommend that further clinical evidence-based studies with a large sample size and prospective design be conducted to check the validity and reliability of the proposed examination sieve and risk alarm score.

## 6. Conclusions

Malignant tumors of the maxillary sinus have a very insidious course of spread and uncertain presentation. These tumors usually present late and are associated with specific clinical signs and symptoms that can be used in their early detection and diagnosis. Therefore, clinicians must be aware of these signs and symptoms and should fully investigate patients at the slightest suspicion of a tumor, albeit benign. A checklist pertaining to the clinical signs and symptoms of these tumors is of value in this regard. The examination sieve and a risk alarm score presented in this study will help general medical and dental practitioners to stay vigilant and increase their referral of patients to tertiary care centers.

## Figures and Tables

**Table 1:** Gross clinical signs and symptoms of maxillary sinus tumors in both sexes (*n* = 70).

Sign and Symptoms	Gender	*N*	Total *N* (%)	*p*-Value
Exophthalmos	Male	20	35 (50.0)	0.571
	Female	15		
Loss of sense of smell	Male	18	37 (52.9)	0.391
	Female	19		
Oroantral fistula	Male	20	39 (55.7)	0.683
	Female	19		
Palpable mass in the upper buccal sulcus	Male	30	66 (94.3)	0.287
	Female	36		
Palpable lymph nodes in the neck	Male	14	25 (35.7)	0.189
	Female	11		
Obstruction of the nose	Male	39	67 (95.7)	0.941
	Female	28		
Bleeding from the nose	Male	30	56 (80.0)	0.582
	Female	26		
Asymmetry of the face	Male	39	69 (98.6)	0.382
	Female	30		
Swelling of the cheek	Male	41	69 (98.6)	0.918
	Female	28		
Double vision	Male	14	24 (34.3)	0.118
	Female	10		
Numbness of the cheek and infraorbital skin	Male	23	41 (58.6)	0.372
	Female	18		

**Table 2:** Association of maxillary sinus tumors with a history of hazards exposure (*n* = 70).

History of Hazard Exposure	Final Diagnosis of Pathology					Total (*N*)
	SCC (*N*)	ACC (*N*)	AC (*N*)	GCT (*N*)	RMS (*N*)	
Wood dust	13	9	3	0	1	26
Tobacco smoke exposure (first or secondhand)	22	5	0	0	0	27
Wood and/or cow dung smoke	5	0	2	1	0	8
Coal dust	3	2	1	0	0	6
Spray paint/chromium	3	0	0	0	0	3
Chi-square	1.412	3.035	2.717	2.4764	1.763	-
df	1	4	3	2	6	-
Standard error	0.365	0.231	0.268	0.274	0.194	-
Spearman correlation	0.064	0.078	0.015	0.048	0.018	-
*p*-value	* 0.001	* 0.021	0.725	0.427	0.825	-

SCC, squamous cell carcinoma; ACC, adenoid cystic carcinoma; AC, adenocarcinoma; GCT, granular cell tumor; RMS, rhabdomyosarcoma. * *p* ≤ 0.05 was considered significant.

## Data Availability

The data included in the present study are available upon request from corresponding author.

## Supplementary Materials

The following supporting information can be downloaded at: https://www.mdpi.com/article/10.3390/healthcare11020194/s1, Figure S1. Clinical Examination Sieve for Malignant tumors of Maxillary sinus; Table S1: Malignant tumors of maxillary sinus, symptoms for early Diagnosis and Risk Alarm Score; Annexure S1: Early and late presentation signs and symptoms of Malignant Tumors of the Maxillary Sinus.

## Appendix A

**Table A1:** STROBE Statement—Checklist of items that should be included in reports of cross-sectional studies.

	Item No	Recommendation	
**Title and abstract**	1	(*a*) Indicate the study’s design with a commonly used term in the title or the abstract	√
		(*b*) Provide in the abstract an informative and balanced summary of what was done and what was found	√
**Introduction**			
Background/rationale	2	Explain the scientific background and rationale for the investigation being reported	√
Objectives	3	State specific objectives, including any prespecified hypotheses	√
**Methods**			
Study design	4	Present key elements of study design early in the paper	√
Setting	5	Describe the setting, locations, and relevant dates, including periods of recruitment, exposure, follow-up, and data collection	√
Participants	6	(*a*) Give the eligibility criteria, and the sources and methods of selection of participants	√
Variables	7	Clearly define all outcomes, exposures, predictors, potential confounders, and effect modifiers. Give diagnostic criteria, if applicable	√
Data sources/measurement	8	For each variable of interest, give sources of data and details of methods of assessment (measurement). Describe comparability of assessment methods if there is more than one group	√
Bias	9	Describe any efforts to address potential sources of bias	----------
Study size	10	Explain how the study size was arrived at	√
Quantitative variables	11	Explain how quantitative variables were handled in the analyses. If applicable, describe which groupings were chosen and why	√
Statistical methods	12	(*a*) Describe all statistical methods, including those used to control for confounding	√
		(*b*) Describe any methods used to examine subgroups and interactions	----------
		(*c*) Explain how missing data were addressed	----------
		(*d*) If applicable, describe analytical methods taking account of sampling strategy	√
		(*e*) Describe any sensitivity analyses	√
**Results**			
Participants	13	(a) Report numbers of individuals at each stage of study—e.g., numbers potentially eligible, examined for eligibility, confirmed eligible, included in the study, completing follow-up, and analysed	√
		(b) Give reasons for non-participation at each stage	----------
		(c) Consider use of a flow diagram	----------
Descriptive data	14	(a) Give characteristics of study participants (e.g., demographic, clinical, social) and information on exposures and potential confounders	√
		(b) Indicate number of participants with missing data for each variable of interest	----------
Outcome data	15	Report numbers of outcome events or summary measures	√
Main results	16	(*a*) Give unadjusted estimates and, if applicable, confounder-adjusted estimates and their precision (e.g., 95% confidence interval). Make clear which confounders were adjusted for and why they were included	√
		(*b*) Report category boundaries when continuous variables were categorized	√
		(*c*) If relevant, consider translating estimates of relative risk into absolute risk for a meaningful time period	----------
Other analyses	17	Report other analyses done—e.g., analyses of subgroups and interactions, and sensitivity analyses	√
**Discussion**			
Key results	18	Summarise key results with reference to study objectives	√
Limitations	19	Discuss limitations of the study, taking into account sources of potential bias or imprecision. Discuss both direction and magnitude of any potential bias	√
Interpretation	20	Give a cautious overall interpretation of results considering objectives, limitations, multiplicity of analyses, results from similar studies, and other relevant evidence	√
Generalisability	21	Discuss the generalisability (external validity) of the study results	√
**Other information**			
Funding	22	Give the source of funding and the role of the funders for the present study and, if applicable, for the original study on which the present article is based	√
