# Supplementary material for: The Evaluation of Clinical Signs and Symptoms of Malignant Tumors Involving the Maxillary Sinus: Recommendation of an Examination Sieve and Risk Alarm Score
Source: Healthcare (Basel). 2023 Jan 9;11(2):194. doi: 10.3390/healthcare11020194 (PMC9859382; doi:10.3390/healthcare11020194)
Supplement: Supplementary file 1 [file healthcare-11-00194-s001.zip › Supplementary Annexure S1.pdf]

Title: Early and late presentation signs and symptoms of Malignant Tumors of the Maxillary Sinus

Proforma

Age____ Gender_____ Occupation______________OPD No_______

History of Exposure to Hazard (Risk factor)

1. Wood dust ☐ 2. Tobbaco Smoke exposure first hand or second ☐

3. Wood and or Cow Dung smoke ☐ 4. Coal Dust ☐ 5. Spray paint /chromium ☐

Earliest or initial presentation signs and symptoms noticed by the patient

1. Unilateral Nasal Blockage ☐ 2. Loss of Sense of Smell on one nostril ☐

3. Unilateral lacrimation ☐ 4. Unilateral Cheek Parasthesia ☐ 5. Unlateral Epistaxis ☐

6. Unilateral Maxillary dentition mobility or exfoliation ☐

7. Unilateral Numbness of Maxillary teeth segment ☐

Clinical presentation at the time of diagnosis by the clinician

1. Exophthalmos ☐ 2. Loss of Sense of Smell ☐

3. Communication between oral cavity and Maxillary Sinus ☐

4. Palpable mass in the upper Buccal Sulcus ☐ 5. Palpable Lymph nodes in the neck ☐

6. Obstruction of nose ☐ 7. Bleeding from the nose ☐ 8. Facialasymmetry ☐

9. Cheekswelling ☐ 10. Diplopia ☐ 11. Parasthesia ☐

Final Diagnosis of Pathology based on histopathological report __________________________
