# Supplementary material for: The Evaluation of Clinical Signs and Symptoms of Malignant Tumors Involving the Maxillary Sinus: Recommendation of an Examination Sieve and Risk Alarm Score
Source: Healthcare (Basel). 2023 Jan 9;11(2):194. doi: 10.3390/healthcare11020194 (PMC9859382; doi:10.3390/healthcare11020194)
Supplement: Supplementary file 1 [file healthcare-11-00194-s001.zip › Supplementary Table S1.pdf]

Supplementary Table S1. Malignant tumors of maxillary sinus, symptoms for early Diagnosis and Risk Alarm Score.

Clinical Symptoms Region	Description of Symptoms	Score
Oral Symptoms	Painless palatal or buccal oral mucosal ulceration	1
	Maxillary alveolar bone or palatal enlargement	1
	Oro-antral communication post hypermobile tooth extraction	1
	Extemporaneous exfoliation sound permanent dentition	1
	Unilateral, unexplained mobility of permanent periodontally healthy teeth	1
	Unexplained and permanent numbness of maxillary dentition	1
Nasal Symptoms	Unilateral nasal obstruction and anosmia	1
	Unilateral bloodstained discharge	1
Orbital Symptoms	Exophthalmos	1
	Increased lacrimation	1
	Diplopia	1
	Proptosis	1
	Recurrent conjunctivitis	1
Neurological Symptoms	Absent and decreased corneal reflex	1
	Infraorbital, cheek paresthesia, or anesthesia	1

This criterion will identify individuals with a risk of suspected MTMS that should prompt dental practitioners to further investigations and subsequent referrals to specialist clinics

Total MTMS score 15, a) 0-5 mild, b) 5-10 moderate, c) 10-15 severe

- Greater than 3 scores in oral symptoms
- Greater than 1 score in nasal symptoms
- Greater than 2 scores in orbital symptoms
- Greater than 1 score in neurological symptoms

Should be considered high risk for MTMS
