# Supplementary figures and images for: The Evaluation of Clinical Signs and Symptoms of Malignant Tumors Involving the Maxillary Sinus: Recommendation of an Examination Sieve and Risk Alarm Score
Source: Healthcare (Basel). 2023 Jan 9;11(2):194. doi: 10.3390/healthcare11020194 (PMC9859382; doi:10.3390/healthcare11020194)

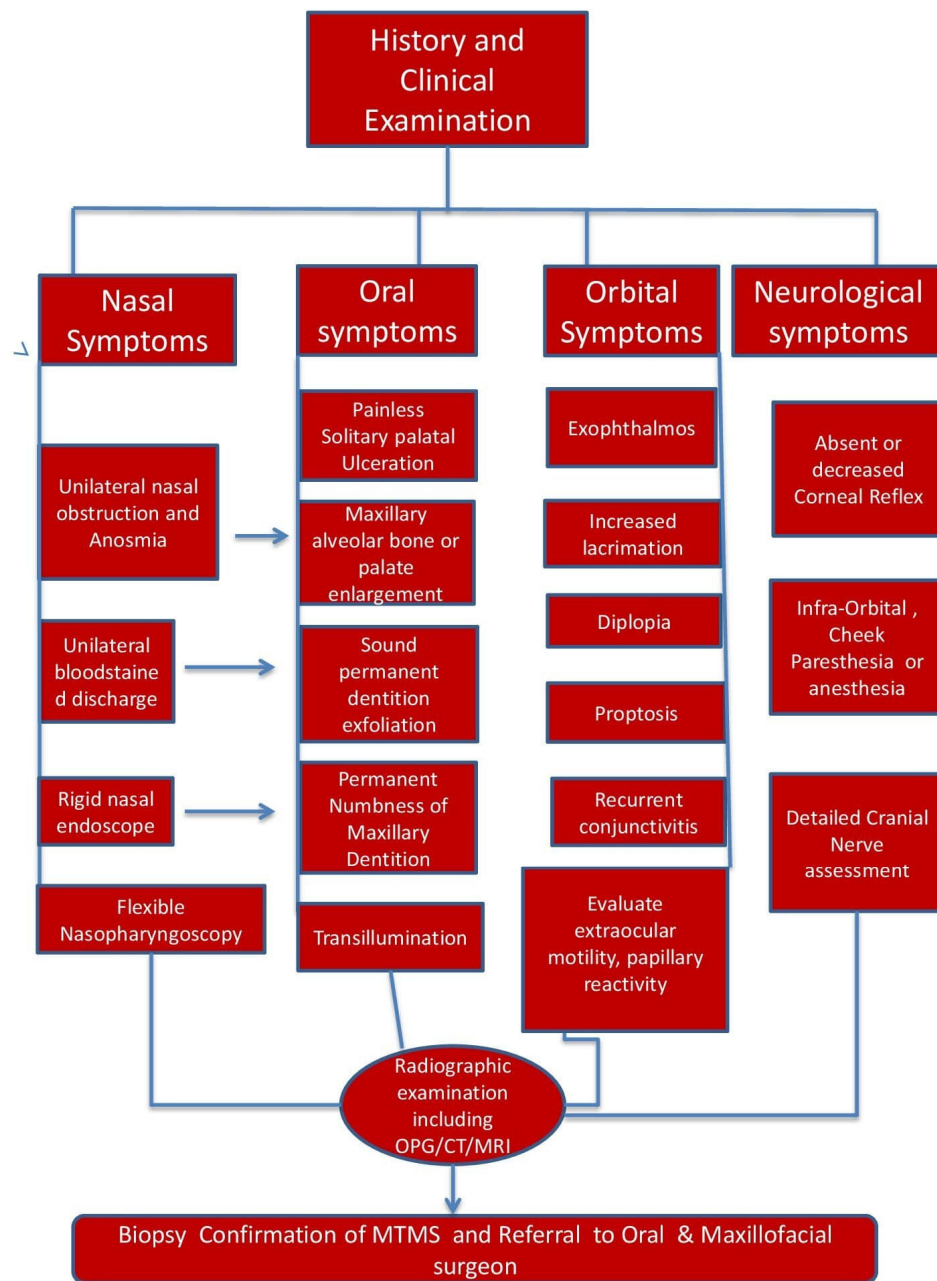

**Supplementary Figure S1.** Clinical Examination Sieve for Malignant tumors of Maxillary sinus.

Supplement: Supplementary file 1 [file healthcare-11-00194-s001.zip › Supplementary Figure S1.pdf]
